# The Essential Role of Executive Attention in Unconscious Visuomotor Priming

**DOI:** 10.3389/fpsyg.2022.800781

**Published:** 2022-05-30

**Authors:** Xuechen Mao, Chun Xie, Jilong Shi, Qin Huang, Ruichen Jiang, Fanying Meng, Hejun Shen, Lyufeng Miao, Shuchen Cui, Anmin Li

**Affiliations:** ^1^School of Psychology, Shanghai University of Sport, Shanghai, China; ^2^Department of Physical Education, Shanghai Jiao Tong University, Shanghai, China; ^3^School of Teacher Education, Anqing Normal University, Anqing, China; ^4^Institute of Physical Education, Huzhou University, Huzhou, China; ^5^Graduate Department, Nanjing Sport Institute, Nanjing, China; ^6^College of Sport Training, Nanjing Sport Institute, Nanjing, China; ^7^International Department, Jinling High School Hexi Campus, Nanjing, China

**Keywords:** executive attention, central executive load, unconscious visuomotor priming, subliminal visual processing, N-back

## Abstract

Many reports have emphasized that unconscious processing demands attention. However, some studies were unable to observe a modulation of attentional load in subliminal visual processing. We proposed that the paradoxical phenomena could be explained based on whether the mental workload task was involved in central executive processes. In two experiments, by combining a masked shape discrimination task with an N-back task, executive attention availability for masked visuomotor processing decreased as the N-back task demand increased. We observed that unconscious visuomotor priming diminished with increasing executive attention load in Experiment 2; however, this pattern did not occur in Experiment 1. Further analysis verified that in Experiment 1, the role of the central executive in unconscious visuomotor priming was eliminated by the accuracy-speed trade-off since the higher load spatial N-back tasks with larger memory set sizes, compared with higher load verbal N-bask tasks, were quite difficult for the subjects to manage. Therefore, our results demonstrated that central executive load modulates unconscious visuomotor priming and that this modulation can be weakened by task difficulty. Collectively, by emphasizing the essential role of executive attention in subliminal visuomotor priming, the present work provides a powerful interpretation of prior debates and develops extant attention capacity limitations from the realm of consciousness to that of unconsciousness.

## Introduction

Unconsciously presented stimuli can shorten response times (RTs) associated with the target when the stimuli share the same response with the target (congruent condition) compared with when the stimuli and the target have different responses (incongruent condition), which is defined as the unconscious visuomotor priming effect (Ulrich and Kiefer, 2016). Based on classic automaticity theories, different from conscious processing, which is widely considered as a type of controlled processing, unconscious processing is triggered involuntarily in a bottom-up manner and is independent of attention resources (Posner and Snyder, 1975; Schneider and Shiffrin, 1977; Jack and Shallice, 2001). As a type of unconscious processing, unconscious visuomotor priming is therefore traditionally thought to operate autonomously and is not amenable to attentional top-down modulation. In line with this view, some researchers hold that unconscious visuomotor priming does not vary when observers are involved in making multiple decisions (Perea et al., 2018).

Over the last two decades, however, many studies have countered this view by providing evidence that subliminal visual processing is influenced by temporal attention (Schubert et al., 2013), spatial attention (Mastropasqua and Turatto, 2015), task sets (Kiefer, 2019), and mental workload (Hung et al., 2020). For instance, by introducing a double Stroop paradigm with a continuous flash suppression method, where an invisible colored prime (e.g., the word “RED” in blue) was followed by a visible colored target (e.g., the word “BLUE” in red) and participants were instructed to respond to the target, Hung et al. (2020) found that a significant unconscious priming effect was only observed when the participants performed the task under a low-load condition but not when the participants performed the task under a high-load condition. They considered their results evidence for the dependence of unconscious priming on attentional load.

However, some studies observed neither the modulation of attentional load on subliminal priming (e.g., Perea et al., 2018) nor the modulation of attentional load on visuomotor priming (e.g., Rajsic et al., 2020). In these studies, participants completed a priming task while performing a memory maintenance task, and the concurrent resource demanding task did not interfere with the priming effect. These studies seemed to support the view that subliminal visuomotor priming was essentially automatic and was immune to attentional resources. However, one should note that attention resources are not monolithic (Chun et al., 2011) and that attention has various resource pools (Cohen et al., 2012). Hence, when subliminal visuomotor priming was observed not to interfere with a concurrent memory maintenance task, we cannot simply conclude that subliminal visuomotor priming was independent of attention resources because we cannot rule out the possibility that the two processing tasks depend on separate attention resources.

Based on the multicomponent model of working memory, limited attention resources are devoted to the maintenance and manipulation of information (Baddeley, 2012). Although these two types of information processing are tightly interlinked, they share quite different resource pools (Miller et al., 2018). Different from maintenance processing, which temporarily stores verbal and visuospatial information, manipulation processing involves the online monitoring, updating, and manipulating of information; thus, the latter needs executive attention (Baddeley, 2012). Notably, previous studies claimed that visuomotor priming was not amenable to attention, and memory maintenance tasks instead of memory manipulation tasks were deployed (Perea et al., 2018; Rajsic et al., 2020). Although it is likely that subliminal priming or visuomotor priming is immune to attention, one possible reason for these results is that their experimental designs do not effectively manipulate the executive attention load, which might contribute to subliminal visuomotor priming (Ansorge et al., 2014). However, there have been few investigations into the effect of executive attention on subliminal visuomotor priming; therefore, the current work aims to address this issue.

In addition, it should be noted that the stimuli domain is a critical factor in this line of work (Ahn et al., 2017). Martens et al. (2011) introduced a special dual-task paradigm in which an induction task was followed by a masked priming task and the two tasks shared the same or different stimuli domain (semantic-semantic vs. perceptual-semantic vs. perceptual-perceptual vs. perceptual-semantic). The behavioral and electrophysiological results showed that a significant unconscious semantic priming effect was observed when the unconscious semantic task followed a semantic induction task but not when the unconscious semantic task followed a perceptual induction task; correspondingly, a significant unconscious visuomotor priming effect was observed when the unconscious visuomotor task followed a perceptual induction task but not when the unconscious visuomotor task followed a semantic induction task. The authors concluded that when an induction task shares the same stimuli domain with the subsequent unconscious priming task, even though the two tasks are quite different, the former will facilitate the latter; hence, unconscious priming was facilitated. Furthermore, this type of facilitation induced by the stimuli domain survives regardless of the variation in the induction task approach (Kiefer, 2019). Therefore, whether the type of stimuli presentation involved in the load task mediates the effect of executive attention on unconscious visuomotor priming deserves to be explored.

The modulation of executive attention on subliminal visuomotor priming was assessed by measuring the variation in subliminal visuomotor priming under different executive attention loads. This condition was created by combining the masked shape discrimination task with the N-back task, the latter is an effective method to vary executive attention loads. We predicted that if masked visuomotor priming depended on executive attention, it would dwindle with incremental increases in central executive load. That is, from the 1-back to the 3-back task, the RT difference between congruent and incongruent conditions in the masked shape decision task should gradually attenuate. Considering that the modulation of executive attention on mask visuomotor priming could be affected by the stimuli domain (i.e., spatial, verbal) of the N-back task, we ran two experiments (a spatial N-back task interleaved with a masked visuomotor priming task in Experiment 1 and a verbal N-back task interleaved with a masked visuomotor priming task in Experiment 2) to investigate this topic. We predicted that if the stimuli domain influenced the effect of executive attention on unconscious visuomotor priming, we would observe distinct results in Experiments 1 and 2. That is, a significant decrease in unconscious visuomotor priming with an increment in the workload of the verbal N-back task should be observed in Experiment 1 since the dual-task paradigm has a different stimuli domain, whereas this pattern should not be observed in Experiment 2 since the dual-task paradigm has a similar stimuli domain.

## Materials and Methods

### Participants

Considering that 24 participants were sufficient to achieve a power of 0.8 in an *F* test, given α = 0.05 (Ducrocq et al., 2017), we recruited 68 college students [34 in each experiment, 40 males, mean (SD) age, 21.69 (2.74) years] to participate in our study. They were right-handed, with normal or corrected-to-normal vision. The Ethical Committee of Shanghai University of Sport (#102772021RT020) approved this research.

## Experiment 1

### Stimuli and Apparatus

On the basis of previous work (Geng et al., 2020), two geometries (vertical x horizontal: ellipse, 2.0 x 4.0°, diamond, 4.0 x 2.0°) were utilized as primes and targets in the masked shape identification task. The masks (4.0 x 4.0°) were composed of many randomly oriented lines. The stimuli for the N-back task consisted of 3 x 4 light gray rectangles (4.0 x 4.0°), one of which was randomly solid black (12 memory items in total). All the stimuli were white and were presented on a dark gray background a viewing distance of 60 cm, (resolution = 1,400 × 900 pixels, frame duration = 16.67 ms, and frequency = 60 Hz). The E-prime 2.0 software package (Schneider et al., 2002) was used in our research.

### Design and Procedure

**Figure 1:**
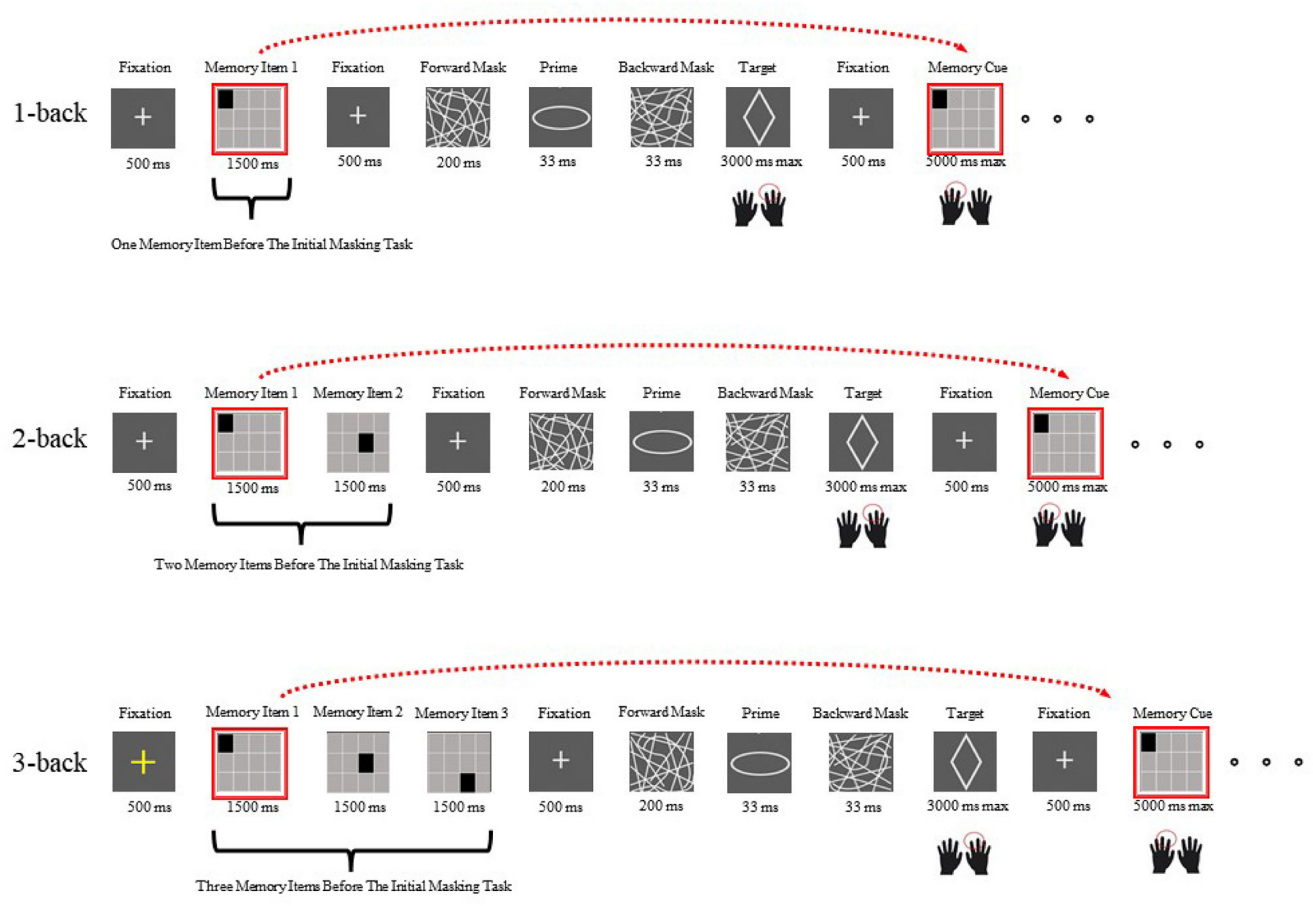
Examples of the first N-back task interleaved with the initial masked visuomotor priming task under each load condition in Experiment 1 (the top row shows the 1-back condition, the middle row shows the 2-back condition and the bottom row shows the 3-back condition). The only variation was that in Experiment 2, the memory items were digits.

Each block started with a 500 ms yellow fixation cross, followed by an initial memory set (one memory item presented for 1,500 ms under the 1-back condition, two memory items presented in sequence for a total of 3,000 ms under the 2-back condition, and three memory items presented sequentially for a total of 4,500 ms under the 3-back condition). The participants needed to continually hold the memory set in mind because after a masked shape decision task was completed, they were asked to recall whether the currently observed memory probe was the same as the one observed one (1-back), two (2-back), or three (3-back) trials prior (Figure 1). After a white fixation dot (500 ms) was presented, a prime was presented for 33 ms, which was masked by forward (200 ms) and backward (33 ms) masks. Then, a target appeared, and the participants were asked to report their responses with their right hands (right arrow for an ellipse and left arrow for a diamond) as correctly and quickly as possible. After a response was recorded or 3,000 ms had passed, a white fixation dot was shown for 500 ms, which was replaced by a memory probe. The probe could either be the same as a memory item presented one, two or three steps earlier or not (equal opportunity). Participants were asked to provide their best responses with their left hands while updating their memory sets, since the memory probe would become the new memory item, and would be recalled one, two, or three masked priming tasks later. A response or an interval of 5,000 ms triggered the next masked priming task. Each block comprised four different memory items.

The participants practiced 90 shape identification tasks and 90 N-back tasks (30 for each load) first and then completed the main experiment. In the main experiment, nine masked visuomotor priming tasks were interleaved with nine N-back tasks in one block. In total, each participant performed 69 dual-task blocks (the first 3 blocks were the practice tasks, and the next 20 blocks were the main tasks for each load condition), including 621 subliminal visuomotor priming tasks and 621 N-back tasks. The order of the load conditions was counterbalanced across the participants. Within each load condition, in half the trials, the prime was congruent with the target while the prime was incongruent for the other half. Additionally, the two shapes appeared equally often as primes and as targets. Additionally, each memory item appeared equally often.

After the main experiment, the participants were informed of the existence of the prime and then performed the objective and subjective prime awareness measures. The sequence of the awareness measures was identical to that of the main experiment except for the following three differences: first, the participants had to identify the shape of the prime when the target appeared; second, the participants were to report their subjective awareness of the prime on a 4-point perceptual awareness scale (PAS: 1 = no experience, 2 = weak glimpse, 3 = almost clear, 4 = absolutely clear); and third, the participants were given enough time to provide a response since accuracy was stressed over speed during this test. Accordingly, the target and memory probe did not disappear until a response was collected. Moreover, only the low-load condition was realized in this part of the study because the suppression effect of the mask weakened at a low load relative to that at a high load (Spinks et al., 2004). The entire awareness task included 12 blocks (the first 2 blocks were practice tasks, and the next 10 blocks were main awareness tasks), consisting of 108 1-back tasks, 108 objective tasks, and 108 subjective tasks.

### Statistical Analysis

Regarding the prime awareness of each observer, *d*' sensitivity measures (Kiefer, 2019; Geng et al., 2020) were assessed from correct responses to congruent trials (hit rates) and erroneous responses to incongruent trials (false alarms). A one-sample *t* test was separately conducted on d' (tested against 0) and objective performance (tested against 0.5). In addition, Pearson's correlation between subliminal priming under the 1-back condition and d' was performed. For the N-back task, a repeated-measures analysis of variance (ANOVA) was performed for the RTs and error rates (ERs). Considering the shape decision task, a 3 (load condition: 1-back vs. 2-back vs. 3-back) x 2 (congruency: congruent vs. incongruent) repeated-measures ANOVA was sequentially conducted for the RTs and ERs.

### Results and Discussion

Six observers were discarded due to their higher ERs (more than 2 standard deviations over the group average); therefore, the remaining 28 observers were included in further analysis.

### Awareness Task

The masking method was effective. None of the observers reported awareness of the prime, and the subjective mean score of the prime was 1.47 (SD = 0.48). Additionally, the ER of the objective measure was close to chance (mean ± standard error of the mean (SEM): 48.78 ± 1.64%), *t*(27) = −0.747, *p* = 0.461. Most importantly, the *d*' measures were not significantly different from zero (−0.05 ± 0.05), *t* (27) = −0.936, *p* = 0.358. Furthermore, the distribution of *d*' and subliminal visuomotor priming was normal (Kolmogorov–Smirnov = 0.113, *p* = 0.20, Kolmogorov–Smirnov = 0.107, *p* = 0.20, respectively), and *d*' and subliminal visuomotor priming did not correlate with each other (*r* (28) = 0.128, *p* = 0.517).

### N-Back Task

Central executive load manipulation was efficacious. Trials with RTs two standard deviations below or above the individual mean were rejected from the analysis (1-back: 1.5%, 2-back: 4.5%, and 3-back: 5.5%). For the RTs, a significant main effect of load condition was observed, *F* (2,26) = 15.318, *p* < 0.001, $\eta_{\text{p}}^{2}$ = 0.362, suggesting that the RTs of 1-back tasks (958.7 ± 35.9 ms) were always smaller than the RTs of 2-back tasks (1,083.05 ± 57.46 ms), and the latter were always smaller than the RTs of 3-back tasks (1,252.5 ± 70 ms) (*p* = 0.01, *p* = 0.002, sequentially). For the ERs, the main effect of load was also significant, *F* (2,26) = 20.126, *p* < 0.001, $\eta_{\text{p}}^{2}$ = 0.427, indicating that the ERs of 1-back tasks (1.93 ± 0.3%) were always smaller than the ERs of 2-back tasks (5.64 ± 0.9%), and the latter were always smaller than the ERs of 3-back tasks (7.73 ± 1.04%) (*p* < 0.001, *p* = 0.025, respectively).

### Masked Visuomotor Priming Task

**Figure 2:**
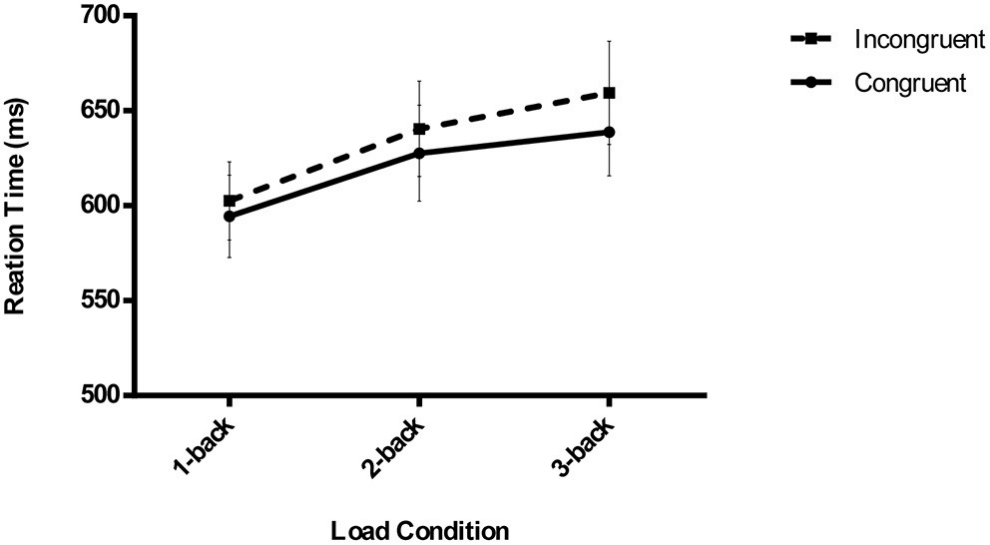
Mean reaction times of unconscious visuomotor priming are depicted as a function of executive attention load (1-back vs. 2-back vs. 3-back) and congruency conditions (congruent vs. incongruent) in Experiment 1. The error bars denote SEM.

The outlier criterion was identical to that of the N-back task (1-back: 3.55%, 2-back: 4.42%, and 3-back: 5.85%). The results demonstrated that subliminal visuomotor priming was significant, *F* (1,27) = 17.365, *p* < 0.001, $\eta_{\text{p}}^{2}$ = 0.391, showing that the RTs for the congruent trials (620.31 ± 21.99 ms) were significantly shorter than those for the incongruent trials (634.14 ± 22.6 ms). Additionally, there was a significant main effect of load, *F* (2,26) = 6.303, *p* = 0.003, $\eta_{\text{p}}^{2}$ = 0.189, and an analysis of the simple effect showed that the RTs of 1-back tasks (598.47 ± 21.03 ms) were always lower than those of 2-back (634.08 ± 25.06 ms) and 3-back tasks (649.13 ± 25.07 ms) (*p* = 0.018, *p* = 0.003, respectively). However, there was no interaction between congruency and load conditions, *F* (2,26) = 1.375, *p* = 0.262, $\eta_{\text{p}}^{2}$ = 0.048, implying that subliminal priming did not significantly decrease with increasing executive attention load (congruent vs. incongruent, 1-back: 594.43 ± 21.66 vs. 602.51 ± 20.63 ms, 2-back: 627.66 ± 25.28 vs. 640.49 ± 25.14 ms, and 3-back: 638.83 ± 23.13 vs. 659.42 ± 27.23 ms) (Figure 2 shows the pattern of the results of the RTs of the masked visuomotor priming task in Experiment 1).

An analogous analysis of the ERs showed a main effect of load, *F* (2,26) = 3.507, *p* = 0.037, $\eta_{\text{p}}^{2}$ = 0.155; the simple effect suggested that the ERs of 1-back tasks (0.7 ± 0.1%) were significantly smaller than those of 3-back tasks (1.2 ± 0.2%) (*p* = 0.006), and neither the difference in ERs between 1-back and 2-back tasks (1 ± 0.2%), nor that between 2-back and 3-back tasks was significant (*p* = 0.113, *p* = 0.385, respectively). Moreover, neither a main effect of congruency, *F*(1,27) = 0.005, *p* = 0.942, $\eta_{\text{p}}^{2}$ = 0.000, nor an interaction was found (congruent vs. incongruent, 1-back: 0.6 ± 0.2 vs. 0.83 ± 0.2%, 2-back: 0.91 ± 0.2 vs. 1.11 ± 0.3%, and 3-back: 1.34 ± 0.3 vs. 0.99 ± 0.2%), *F* (2,26) = 1.704, *p* = 0.192, $\eta_{\text{p}}^{2}$ = 0.059 (Supplementary Figure 1).

In summary, the results of Experiment 1 did not show the modulation of executive attention load on unconscious visuomotor priming. It is important to stress that Ozimič and Repovš (2020) proposed that visual working memory capacity was confined by a visual representational system, which could overlap with the representational system of subliminal visuomotor stimuli (Carlisle and Kristjánsson, 2018; Persuh et al., 2018) and consequentially weaken the modulation of executive attention on visuomotor priming. Specifically, based on the attention sensitivity model (Martens et al., 2011; Kiefer, 2019), thte spatial N-back task shares a similar stimuli domain with the unconscious visuomotor priming task; the former might facilitate the subsequent processing of masked priming, thereby compensating for the inhibition of the processing of unconscious visuomotor priming from the loaded spatial N-back task; hence, unconscious visuomotor priming remains invariant or even slightly increased instead of being eliminated, and the effect of executive attention on unconscious visuomotor priming vanishes. Ulrich et al. (2014) introduced functional magnetic resonance imaging (fMRI) with a dual-task protocol in which a semantic or a perceptual induction was followed by an unconscious lexical priming task and found that greater functional connectivity with the ventral occipitotemporal area of the brain (this brain area is engaged in semantic processing) after the semantic induction task relative to the perceptual induction task. This neural pattern is strongly related to the magnitude of behavioral and neural priming. Therefore, when following the spatial N-back task, unconscious visuomotor priming was facilitated rather than disrupted.

By combining verbal memory tasks with semantic priming tasks, an increase in semantic priming was observed with an increase in verbal memory workload (De Loof et al., 2013). Although these findings seem to support the results of Experiment 1, a critical difference in the experimental designs of these two studies needs to be noted. The attention load task deployed in the extant study was a memory maintenance task, while the attention load task deployed in our study was a memory manipulation task. The maintenance task does not occupy executive attention, while the manipulation task does occupy executive attention; thus, executive attention survived with an increase in maintenance workload in that study but was consumed with an increase in maintenance workload in our study. Correspondingly, although all the attention load tasks in these two studies share similar stimuli representations with combined unconscious priming tasks, the unconscious priming effect in the extant study was significantly increased, while the unconscious priming effect in our study was weakly increased.

Considering the influence of the stimuli domain on the modulation of executive attention on unconscious visuomotor priming, the spatial N-back task used in Experiment 1 was changed to the verbal N-back task that was used in Experiment 2 to address these concerns, as the latter was not as affected by visual-spatial representations as the former.

## Experiment 2

### Stimuli, Apparatus and Procedure

The experimental design was the same as that in Experiment 1 except that the memory items switched from rectangles (spatial domain) to digits (verbal domain). That is, the stimuli for the N-back task in Experiment 2 consisted of 10 single digits from zero to nine (1.0 x 0.7°). Each block in the dual-task protocol comprised four different memory items extracted randomly from the 10 digit stimuli, and each digit had an equal opportunity to be presented as a memory item in the verbal N-back task.

### Results and Discussion

Five observers were removed from the sample because of their higher ERs. Thus, we further analyzed the data from the remaining 29 observers.

### Awareness Task

The suppression method was successful. Only one observers, who reported a weak glimpse of some images between the masks but had no idea of the exact content of the images, reported awareness of the prime. Moreover, the mean subjective rate score was 1.37 (SD = 0.38). Additionally, the performance of the objective measure was close to chance (53.61 ± 2.3%), *t* (28) = 1.57, *p* = 0.128. Furthermore, the *d*' values were not significantly different from zero (0.098 ± 0.059), *t* (28) = 1.673, *p* = 0.105. Furthermore, the distribution of *d*' was normal (Kolmogorov–Smirnov = 0.102, *p* = 0.20), while that of subliminal priming under the 1-back task (Kolmogorov–Smirnov = 0.178, *p* = 0.02) was not normal. Therefore, a logarithmic conversion was applied to the magnitude of subliminal priming, and the correlation analysis results showed that subliminal priming was independent of *d*', *t* (29) = −0.125, *p* = 0.542. Notably, there was no significant difference in subjective measures, objective measures, and *d*' between Experiments 1 and 2, where *t* (55) = 0.906, *p* = 0.369, and *t* (55) = −1.702, *p* = 0.094, and *t* (55) = −1.869, *p* = 0.067, respectively.

### N-Back Task

The executive attention load manipulation was effectual. Outliers were rejected in the same manner as in Experiment 1 (1-back: 4.02%, 2-back: 4.59%, and 3-back: 4.86%). For the RTs, we found a significant main effect of load condition, *F* (2,27) = 20.931, *p* < 0.001, $\eta_{\text{p}}^{2}$ = 0.608, suggesting that the RTs of 1-back tasks (852.16 ± 44.66 ms) were always shorter than those of 2-back tasks (1,250.79 ± 76.2 ms), and the latter were always shorter than those of 3-back tasks (1,390.77 ± 100.08 ms) (*p* < 0.001, *p* = 0.042, sequentially). For the ERs, the main effect of load was also significant, *F* (2,27) = 4.212, *p* = 0.02, $\eta_{\text{p}}^{2}$ = 0.131, and the analysis of the simple effect indicated that the ERs of 3-back tasks (4.93 ± 0.8%) were significantly larger than those of 1-back (3.12 ± 0.4%) and 2-back tasks (3.47 ± 0.6%) (*p* = 0.02, *p* = 0.045, respectively).

### Masked Visuomotor Priming Task

**Figure 3:**
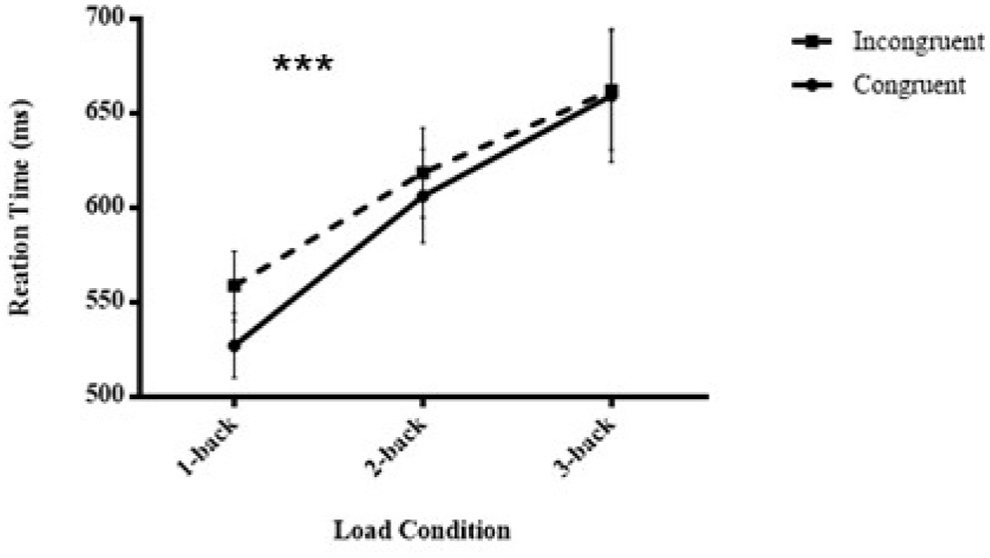
Mean reaction times of unconscious visuomotor priming are illustrated as a function of executive attention load (1-back vs. 2-back vs. 3-back) and congruency conditions (congruent vs. incongruent) in Experiment 2. ***p < 0.001.

Subliminal visuomotor priming was significant (outliers for 1-back tasks: 1.95%, 2-back tasks: 4.5%, and 3-backs: 7.2%), *F* (1,28) = 7.478, *p* = 0.011, $\eta_{\text{p}}^{2}$ = 0.211, showing that the RTs for congruent trials (597.59 ± 24.17 ms) were statistically shorter than those for incongruent trials (613.17 ± 22.89 ms). Additionally, there was a significant main effect of load, *F* (2,27) = 13.773, *p* < 0.001, $\eta_{\text{p}}^{2}$ = 0.505, indicating that the RTs of 1-back tasks (543.17 ± 17.45 ms) were always smaller than the RTs of 2-back tasks (612.45 ± 23.95 ms), and the latter were always smaller than the RTs of 3-back tasks (660.77 ± 33.15 ms) (*p* < 0.001, *p* = 0.006, respectively). Most importantly, there was an interaction between congruency and load condition, *F* (2,27) = 8.808, *p* < 0.001, $\eta_{\text{p}}^{2}$ = 0.239. This result implied that subliminal priming significantly decreased when executive attention load increased (congruent vs. incongruent, 1-back: 527.1 ± 17.09 vs. 558.72 ± 18.34 ms, 2-back: 606.26 ± 24.61 vs. 618.64 ± 23.69 ms, and 3-back: 659.41 ± 35.11 vs.662.14 ± 31.59 ms) (Figure 3 shows the pattern of the results of the RTs of the masked visuomotor priming task in Experiment 2).

An analogous analysis of ERs showed a main effect of load, *F* (2,27) = 4.758, *p* = 0.012, $\eta_{\text{p}}^{2}$ = 0.145, and the simple effect suggested that the ERs of 1-back tasks (0.8 ± 0.2%) were always smaller than those of 2-back (1.7 ± 0.3%) and 3-back tasks (1.4 ± 0.3%) (*p* = 0.007, *p* = 0.05, respectively). However, neither a main effect of congruency, *F* (1,28) = 0.418, *p* = 0.523, $\eta_{\text{p}}^{2}$ = 0.015 nor an interaction was found (congruent vs. incongruent, 1-back: 0.69 ± 0.2 vs. 0.84 ± 0.2%, 2-back: 1.65 ± 0.3 vs. 1.72 ± 0.4%, and 3-back: 1.42 ± 0.3 vs. 1.46 ± 0.4%), *F* (2,27) = 0.027, *p* = 0.974, $\eta_{\text{p}}^{2}$ = 0.001 (Supplementary Figure 2, Table 1).

**Table 1:** Mean reaction times (RTs in milliseconds), and error rates (ERs in percentage) as a function of executive attention load (1-back vs.2-back vs.3-back), and congruency conditions (congruent vs. incongruent) in Experiments 1 and 2.

			**Experiment 1**			**Experiment 2**		
			**1-back**	**2-back**	**3-back**	**1-back**	**2-back**	**3-back**
EALT	RT		958.7 (35.9)	1,083.05 (57.46)	1252.5 (70)	852.16 (44.66)	1,250.79 (76.2)	1,390.77 (100.08)
	ER		1.93 (0.3)	5.64 (0.9)	7.73 (1.04)	3.12 (0.4)	3.47 (0.6)	4.93 (0.8)
UVPT	RT	C	594.43 (21.66)	627.66 (25.28)	638.83 (23.13)	527.10 (17.09)	606.26 (24.61)	659.41 (35.11)
		I	602.51 (20.63)	640.49 (25.14)	659.42 (27.23)	558.72 (18.34)	618.64 (23.69)	662.14 (31.59)
	ER	C	0.6 (0.2)	0.91 (0.2)	1.34 (0.3)	0.69 (0.2)	1.65 (0.3)	1.42 (0.3)
		I	0.83 (0.2)	1.11 (0.3)	0.99 (0.2)	0.84 (0.2)	1.72 (0.4)	1.46 (0.4)

*EALT, executive attention load task; UVPT, unconscious visuomotor priming task; RT, reaction time; ER, error rate; C, congruent condition; I, incongruent condition. Standard error in brackets*.

Taken together, in accordance with our hypothesis, the results revealed the effect of executive attention load on subliminal visuomotor priming. That is, an increased executive attention load decreased the size of unconscious visuomotor priming effects. According to the resource limitations of attention (Chun et al., 2011), a task with increased workload will interfere with the performance of another task if the two tasks share a common attention resource. Thus, our results indicated that unconscious visuomotor priming requires attention resource engagement, and the unconscious visuomotor priming processing shares a common attention resource with the N-back task. Considering that the N-back task is a classic executive attention demanding task that is involved in online monitoring, refreshing, and manipulating information, we can conclude that unconscious visuomotor priming also demands executive attention engagement. Hence, a significant unconscious visuomotor priming effect was observed when the N-back task under a lower load condition (there were enough attention resources that could be engaged in masked visuomotor priming processing), and the unconscious visuomotor priming effect decreased or even vanished with the increasing workload of the N-back task (the availability of attention resources for masked visuomotor priming processing were depleted).

It should be noted that the results of Experiment 2 were inconsistent with those of Experiment 1; thus, we are uncertain whether the modulation of executive attention on unconscious visual processing occurs in all cases. However, the stimuli domain of the dual-task paradigm in Experiment 1 was quite different from that in Experiment 2, i.e., a spatial N-back task was combined with an unconscious shape priming task in Experiment 1, while a verbal N-back task was combined with an unconscious shape priming task in Experiment 2. Hence, it is the stimuli representation of the N-back task in Experiment 1 that overlapped with the unconscious visuomotor priming task, rather than the stimuli representation of the N-back task in Experiment 2. Because of the attention sensitivity model (Martens et al., 2011; Ulrich et al., 2014; Kiefer, 2019), when a former task shares a similar stimuli domain with the subsequent unconscious priming task, unconscious priming will be facilitated. Therefore, the facilitation effect elicited by similar stimuli representation between the N-back task and the unconscious visuomotor priming task occurred in Experiment 1 but not in Experiment 2. This facilitation effect attenuated the effect of executive attention load on the unconscious priming effects, which might explain the disparate results between the two experiments. Moreover, the pattern of the results tended to indicate the dependence of unconscious visuomotor priming on executive attention.

## General Discussion

Our goal was to examine whether executive attention modulated unconscious visuomotor priming. Hence, a dual-task paradigm was utilized, where a masked shape decision task was intermixed with an N-back task, and the latter varied only with respect to the stimuli in the two experiments. The results indicated that the magnitude of the unconscious visuomotor priming effect weakened or even disappeared with an increase in executive attention load when the dual-task design shared a different stimuli domain. However, this modulatory effect of central executive load on unconscious visuomotor priming was eliminated when the dual-task design shared the same stimuli domain. Therefore, our results demonstrated that the central executive load modulates unconscious visuomotor priming and that this modulation can be impaired by the stimuli type involved in these processes.

One possibility for the mechanism underlying the modulation of executive attention on unconscious visuomotor priming is that executive processes weaken activity in the primary visual cortex (V1), which is necessary for the processing of unseen visuomotor stimuli. Hurme et al. (2019) examined the contribution of V1 to subliminal visual processing by means of transcranial magnetic stimulation (TMS) and showed that subliminal processing was abolished when TMS interfered with V1 function. Moreover, Bahrami et al. (2007) explored the effect of mental workload on stimuli that individuals were not aware of. In their study, invisible tool images were presented to the subjects, and subjects concurrently completed tasks with low and high difficulty. They clarified that the V1 blood oxygen level-dependent (BOLD) responses produced by subliminal stimuli decreased with an increase in task difficulty. Additionally, in the research of Spinks et al. (2004), as executive load increased, the V1 activation elicited by unexpected stimuli vanished. Thus, it is likely that executive attention influences unconscious visuomotor priming by manipulating V1 activation.

A second possibility is that central executive engagement suppresses the temporoparietal junction (TPJ), which is a crucial coupling for subliminal visuomotor priming. Ulrich and Kiefer (2016) investigated the neural signature of masked visuomotor processing by fMRI. The results indicated that the functional connectivity associated with congruent relative to incongruent trials between ventral visual stream areas and posterior parietal areas, as well as other associated areas, increased. Furthermore, Todd et al. (2005) ran a series of experiments and observed that TPJ activity was suppressed during visual working memory tasks; furthermore, the subsequent processing of 60 ms stimuli was impaired. Hence, the mechanism by which executive load affects unconscious visuomotor priming is supposedly related to TPJ suppression.

Alternatively, executive load and subliminal visuomotor processing compete for a common network, which creates this modulation phenomenon. Based on the research of Ulrich and Kiefer (2016), activity under incongruent conditions was greater than that under congruent conditions in the bilateral inferior and medial superior frontal gyri and other related regions. Additionally, an fMRI study by Bergström and Eriksson (2018) demonstrated that invisible visual processing depended on activity in the frontal and occipital cortex. Moreover, Tomasi et al. (2007) reanalyzed prior fMRI datasets and concluded that executive tasks (N-back) mainly elicited activity in frontal areas in addition to the cortices that overlapped with simple attention demanding tasks.

Although our findings are inconsistent with those of Rajsic et al. (2020) and Perea et al. (2018), there were some differences between the experimental design of the present study and that of their work, which can account for the contradictory results. Most noteworthy, the simultaneous mental workload tasks in their paper were only related to the maintenance load, which did not include the central executive load. According to the work of Tomasi et al. (2007), executive attention load shares different and larger activation patterns than general attention load shares. Additionally, a visuomotor prime was presented visibly to the participants in the research of Rajsic et al. (2020); therefore, their results cannot be simply compared to those of our study.

Albeit indirectly, the findings of many previous reports are in line with our results. In two event-related potential (ERP) experiments, Zovko and Kiefer (2013) indicated that unseen visuomotor processing was influenced by the preceding task, which manipulated the attentional control required for subliminal visuomotor priming. By combining the existing literature about top-down modulation on unconsciousness, Ansorge et al. (2014) summarized several models of executive attention of unconscious visuomotor priming and suggested that unconscious visuomotor processing relied on task-control representations. Nonetheless, few empirical studies have focused on the key role of executive attention in subliminal visuomotor priming; hence, our work goes beyond prior studies insofar that we directly provided evidence to support this view.

Finally, the current findings should be treated with caution. First, different stimuli representations of the dual-task protocol of Experiment 1 and that of Experiment 2 were an important factor resulting in the different results observed in the two Experiments; however, some other interpretations may exist and were not further investigated and discussed in our study. For instance, although the accuracy of the N-back task does not suffer much in Experiment 1 compared to Experiment 2, it should be noted that in the 1-back task, the RTs in Experiment 1 were longer than those in Experiment 2, while in the 2-back and 3-back tasks, the RTs in Experiment 1 were not longer than those in Experiment 2. Accordingly, in the 1-back task, the ERs in Experiment 1 were below those observed in Experiment 2, whereas in the 2-back and 3-back tasks, the ERs in Experiment 1 were above those in Experiment 2. It is possible that the divergent results between the two experiments stemmed from other important reasons, such as the accuracy-speed trade-off caused by task difficulty, which could influence the modulation of executive attention on masked visuomotor processing. Thus, future research could address these matters. Notably, although all the network mechanisms mentioned above are plausible, the true neural patterns behind the modulatory effect of executive attention on unconscious visuomotor priming await further exploration.

## Conclusion

In summary, our results indicate that executive attention availability is necessary for unconscious visuomotor priming. Specifically, the magnitude of the unconscious visuomotor priming effect decreases or even disappears as the central executive load increases. The current evidence solves hitherto conflicting findings by proposing the determining role of the central executive in unconscious visuomotor processing. Furthermore, the present article extends existing frameworks of attention gating systems (Cohen et al., 2012) from the realm of consciousness to that of unconsciousness as well as from the realm of general attention to central executive attention.

## Data Availability Statement

The raw data supporting the conclusions of this article will be made available by the authors, without undue reservation.

## Ethics Statement

The studies involving human participants were reviewed and approved by the Ethics Committee of Shanghai University of Sport (No. 102772021RT020). The patients/participants provided their written informed consent to participate in this study.

## Author Contributions

XM and AL: conceptualization. XM, JS, and HS: methodology. XM, QH, and LM: formal analysis. XM, RJ, FM, and SC: investigation. XM and CX: writing. AL: funding acquisition. All authors contributed to the article and approved the final version of the manuscript for submission.

## Funding

This research was supported by Innovative Research Group Project of the National Natural Science Foundation of China (Grant Numbers: 31971023).

## Conflict of Interest

The authors declare that the research was conducted in the absence of any commercial or financial relationships that could be construed as a potential conflict of interest.

## Publisher's Note

All claims expressed in this article are solely those of the authors and do not necessarily represent those of their affiliated organizations, or those of the publisher, the editors and the reviewers. Any product that may be evaluated in this article, or claim that may be made by its manufacturer, is not guaranteed or endorsed by the publisher.
