# Supplementary material for: The Essential Role of Executive Attention in Unconscious Visuomotor Priming
Source: Front Psychol. 2022 May 30;13:800781. doi: 10.3389/fpsyg.2022.800781 (PMC9198630; doi:10.3389/fpsyg.2022.800781)
Supplement: Supplementary file 1 [file Image_1.pdf]

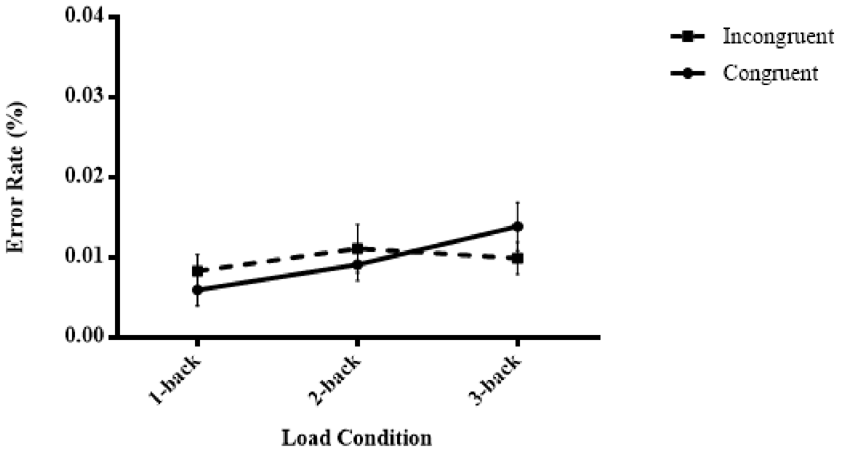

Supplementary Figure 1. Error rate of unconscious visuomotor priming are presented as a function of executive attention load (1-back versus 2-back versus 3-back) and congruency conditions (congruent versus incongruent) in Experiment 1.
